# Industry-scale application and evaluation of deep learning for drug target prediction

**DOI:** 10.1186/s13321-020-00428-5

**Published:** 2020-04-19

**Authors:** Noé Sturm, Andreas Mayr, Thanh Le Van, Vladimir Chupakhin, Hugo Ceulemans, Joerg Wegner, Jose-Felipe Golib-Dzib, Nina Jeliazkova, Yves Vandriessche, Stanislav Böhm, Vojtech Cima, Jan Martinovic, Nigel Greene, Tom Vander Aa, Thomas J. Ashby, Sepp Hochreiter, Ola Engkvist, Günter Klambauer, Hongming Chen

**Affiliations:** 1 Clinical Pharmacology and Safety Science, R&D BioPharmaceuticals, AstraZeneca, Pepparedsleden 1, 43183 Mölndal, Sweden; 2 LIT AI Lab & Institute for Machine Learning, Johannes Kepler University Linz, Altenberger Str. 69, 4040 Linz, Austria; 3 High-Dimensional Biology & Discovery Data Sciences, Discovery Sciences, Janssen Pharmaceutica, Turnhoutseweg 30, 2349 Beerse, Belgium; 4 High-Dimensional Biology & Discovery Data Sciences, Discovery Sciences, Janssen R&D, 1400 McKean Rd, Spring House, Pennsylvania 19002 USA; 5 High-Dimensional Biology & Discovery Data Sciences, Discovery Sciences, Janssen Cilag SA, Calle Río Jarama, 75A, 45007 Toledo, Spain; 6 Ideaconsult Ltd., 4. Angel Kanchev Str., 1000 Sofia, Bulgaria; 7 Intel Corporation, Data Center Group, Veldkant 31, 2550 Kontich, Belgium; 8 IT4Innovations, VSB – Technical University of Ostrava, 17. Listopadu 2172/15, 70800 Ostrava-Poruba, Czech Republic; 9 Exascience Lab, Imec, Kapeldreef 75, 3001 Louvain, Belgium; 10 Hit Discovery, Discovery Sciences, R&D BioPharmaceuticals, AstraZeneca, Pepparedsleden 1, 43183 Mölndal, Sweden

**Keywords:** QSAR, Deep learning, Machine learning, Structure-based virtual screening, Cheminformatics, Big data, ChEMBL, PubChem, Prospective evaluation, Retrospective evaluation

## Abstract

Artificial intelligence (AI) is undergoing a revolution thanks to the breakthroughs of machine learning algorithms in computer vision, speech recognition, natural language processing and generative modelling. Recent works on publicly available pharmaceutical data showed that AI methods are highly promising for Drug Target prediction. However, the quality of public data might be different than that of industry data due to different labs reporting measurements, different measurement techniques, fewer samples and less diverse and specialized assays. As part of a European funded project (ExCAPE), that brought together expertise from pharmaceutical industry, machine learning, and high-performance computing, we investigated how well machine learning models obtained from public data can be transferred to internal pharmaceutical industry data. Our results show that machine learning models trained on public data can indeed maintain their predictive power to a large degree when applied to industry data. Moreover, we observed that deep learning derived machine learning models outperformed comparable models, which were trained by other machine learning algorithms, when applied to internal pharmaceutical company datasets. To our knowledge, this is the first large-scale study evaluating the potential of machine learning and especially deep learning directly at the level of industry-scale settings and moreover investigating the transferability of publicly learned target prediction models towards industrial bioactivity prediction pipelines.

## Introduction

Artificial intelligence (AI) is evolving fast through algorithmic advances in various application fields, including drug discovery [1–3]. Quantitative structure activity relationship (QSAR) studies constitute one of the key elements of early drug development. The aim is to quantify the biological activities of small molecules as a function of their molecular structures. To this end, typical drug development programs adopt a trial and error strategy in which vast numbers of molecules are tested to measure their biological activity in the presence of a target protein. Over the years, large quantities of QSAR data have been generated. The ChEMBL and PubChem databases [4, 5] are regarded as the major resources for publicly available small molecule bioactivity data, containing more than 15 and 239 million bioactivity data points, respectively. Despite the wealth of data, these databases remain difficult to use due to the heterogeneity of their data. It is important to mention that much of the existing QSAR data remains in-house in pharmaceutical companies.

There has been a long tradition in using such datasets to build predictive QSAR classification and regression models with the help of machine learning algorithms [6]. QSAR models typically either heavily rely on the molecular similarity principle assuming that molecules with similar structures have similar biological activities [7], or they are more feature-based, as e.g. the ChEMBL target prediction tool [8–10]. Based on this assumption, machine learning algorithms fit functions that are capable of mapping small molecule structures to their biological activities, which in turn allows to make predictions on new molecules and to prioritize them. In the most basic form, QSAR models are built on a per-project basis with the objective of predicting the activity of new molecules for one single target protein, thus can also be considered as target prediction methods. Therefore, the modelling task consists of compiling one dataset of molecules with activities on one protein and of utilizing one or several so-called single-task algorithms to build predictive models. Some of the most popular methods are random forest [11] (RF) and support vector machine [12] (SVM) models. A comprehensive overview and review of drug target prediction methods is given by Sydow et al. [13].

With the rise of AI in the era of cheap computation, many novel machine learning algorithms have been applied to various application domains such as online customer recommendation [14], speech recognition [15], computer vision [16–19], natural language processing [20] and generative modelling [21]. Drug target prediction also benefits from these newly developed deep learning techniques [22]. One attractive aspect of deep learning in drug discovery is its versatility. Deep learning algorithms offer the possibility of creating multitask models which particularly suit drug target prediction for a panel of target proteins [23–26]. This technique has been notably successful in two machine learning challenges. Dahl et al. (2014) [27] won the Merck Kaggle challenge by applying a multitask fully connected deep neural network and Mayr et al. [28] won the Tox21 challenge by using a similar approach. However, despite the recent successes of deep learning in competitions, and several studies claiming the superiority of multitask deep learning, there is still the question, how well deep learning models perform in industrial settings with much more data compared to competitions and different data distributions compared to publicly available databases and benchmark datasets. We considered this to be the main research question here and in order to gain insights, we first trained predictive machine learning models using diverse different algorithms on public data and then transferred these models to industrial data and analyzed the performances of these models there.

Typically, the dataset used to train a target prediction model represents its underlying chemical applicability domain. It is therefore highly desirable to use a large-scale public dataset for the study following the idea that “the more data the better”. Fortunately, a few large-scale datasets [29–32] have already been created and used to benchmark target prediction algorithms. Mayr et al. [29] created a large-scale benchmark dataset for drug discovery (the LSC dataset), which they provided to the community for model development and method comparisons. The LSC dataset is based on data from ChEMBL and considered each individual assay as a separate prediction task, which should allow a fair method comparison for predicting experimental measurements. Another larger dataset, that follows similar goals as the LSC dataset, is the ExCAPE-DB dataset [30], also created as part of the ExCAPE project. The data of ExCAPE-DB contains information extracted from both the ChEMBL and the PubChem database, whereas the LSC dataset is based purely on ChEMBL. Another main difference of ExCAPE-DB to the LSC dataset is that in ExCAPE-DB different assays were merged together if they were annotated to measure the effect on the same target. This merging of different assays to the same targets may lead to increased noise (see e.g. [33]), but it allows to directly transfer models learned on public data to company internal pharma data by means of a commonly shared target. For the LSC dataset on the other hand, transferring the model would require an accurate mapping from public to private assays, which would demand overwhelming manual inspection, if it would be possible at all. A further aspect of merging different assays, is, that more training data is available per target and that some measurement noise might be averaged out.

For this study, we decided to use ExCAPE-DB, as it is one of the largest open source benchmark target prediction datasets, and allows to evaluate target prediction models trained on public data on their ability to predict industrial QSAR and thus their potential usability in industrial drug discovery projects. To this end, we evaluate the classification performance of the learned models on two industry-size datasets stemming from two different pharmaceutical companies. To be able to draw comparisons of the obtained prediction performances between public and private databases, we also compute prediction performances on ExCAPE-DB itself.

Regarding the machine learning algorithms, we decided to compare deep learning with gradient boosting, which serves as a representative of ensemble learning based approaches such as RFs. We also compare to a Bayesian matrix factorization approach, that considers the problem of target prediction more from the point of view of a regression problem. We do not include a similarity-based approach such as SVMs or k-nearest-neighbors, since this would have resulted in a high additional computational effort due to the large numbers of compounds of many targets being in the range of tens and hundreds of thousands. It should be noted, that ensemble-based approaches as well as the matrix factorization approach are established machine learning methods in pharmaceutical industry [34, 35].

## Methods

### Used public dataset: ExCAPE-ML

QSAR data from ExCAPE-DB was used to construct the ExCAPE machine learning dataset, which will be referred to as the ExCAPE-ML dataset. ExCAPE-DB is a collection of protein–ligand activity entries compiled from ChEMBL and PubChem containing around 70.8 million data points covering about one million compounds and 1667 targets. The collection includes entries from PubChem screens labeled as inactive. Data points from ChEMBL and PubChem were aggregated together with standardized compound structures, official target gene symbols and standardized, log-transformed activity values (pXC50 values). For classification tasks, we assigned the data points to two classes (i.e. inactive, active) according to their pXC50 values. A compound-target record was defined to be active if it fulfilled the criterion that pXC50 ≥ 6 (activity ≤ 1 µM). The dataset was trimmed down by only keeping targets with at least 300 QSAR data points, including at least 75 active compounds and 75 inactive compounds. This resulted in the ExCAPE-ML dataset, being composed of 955,386 compounds, covering 526 distinct target proteins for a total of 49,316,517 QSAR data points (about 90% sparse) with an overall active to inactive ratio close to 1:100.

Roughly, there are two blocks of targets in ExCAPE-ML: 338 targets are annotated with less than 10,000 compounds and 188 targets are annotated with more than 10,000 compounds out of which 155 targets are annotated with between 100,000 and 468,789 compounds (Additional file 1: Fig. S1). The ratio of active to inactive compounds is much higher for smaller targets (less than 100,000 compounds) than for larger targets. The high imbalance level in those large datasets very likely reflects the presence of high-throughput screening data deposited into PubChem, where hit rates are typically low, whereas the low imbalance level in smaller datasets presumably corresponds to data obtained from lead optimization projects in which the aim is often to optimize chemical series of active compounds and where many of the analogues would also be active. Overall, the full compound-target matrix, i.e. the matrix relating compounds and targets by their activities, is very sparse with about 90% of its elements missing.

The 526 target proteins are associated with diverse target families. We further characterized the targets of ExCAPE-ML using information from ChEMBL, EC numbers [36] and UniProt [37]. Details on the dataset composition are available in Additional file 1: Notes S1.

### Used external industrial test sets

**Fig. 1:**
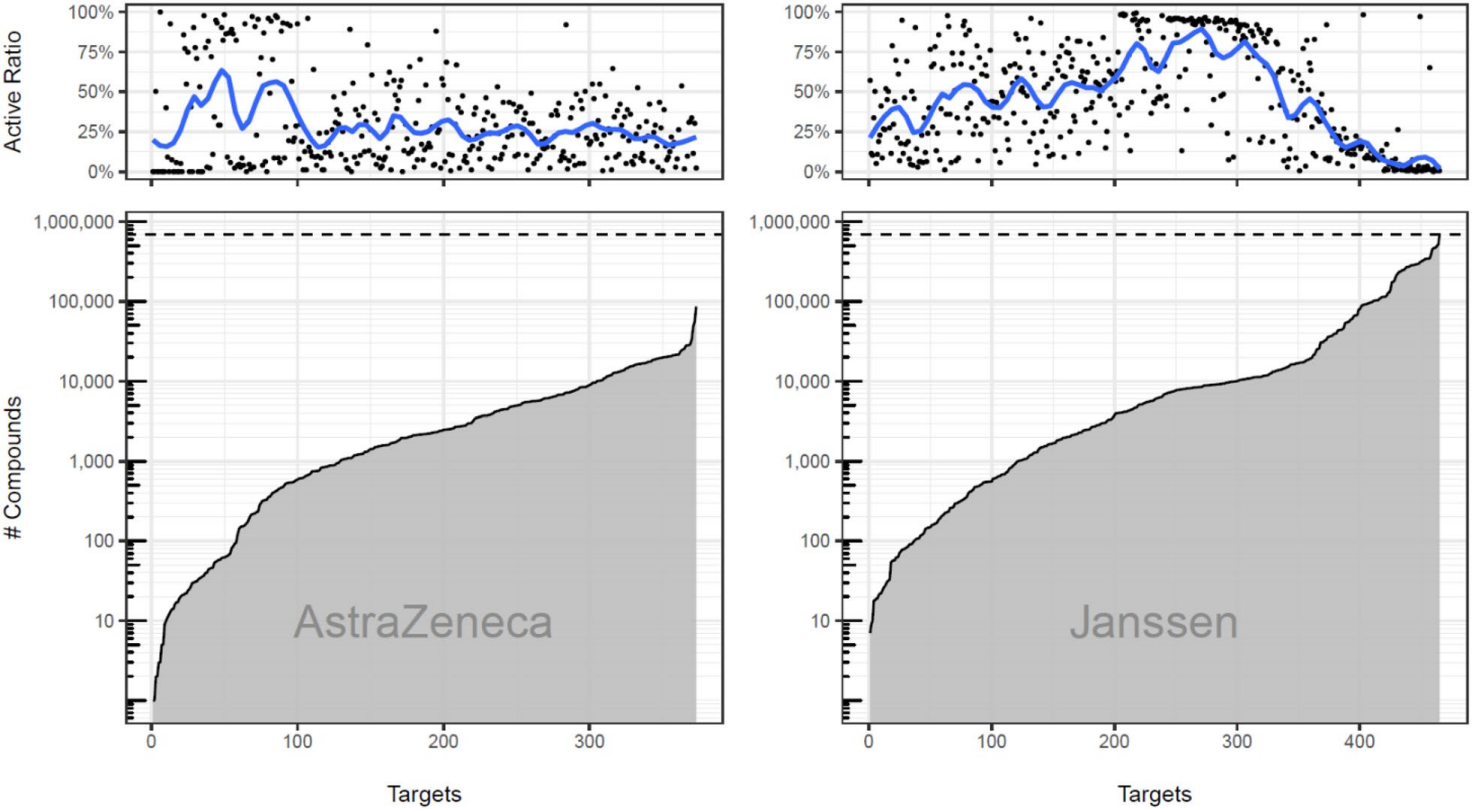
Compound distributions across the targets for the AstraZeneca and the Janssen dataset, respectively. In the lower panel, the y-axis shows the number of compounds for targets represented by the x-axis, where the targets are sorted according to the number of compounds. The horizontal dashed line represents the maximum number of compounds per target observed in the datasets. In the upper panel, a point represents the activity ratio of a target; targets are sorted the same way as in the lower panel. The curve in the upper panel is a smooth average

We built external test sets by querying AstraZeneca and Janssen in-house dose–response screening databases with the 526 targets of ExCAPE-ML. AstraZeneca and Janssen dose–response repositories were queried for assays associated to targets of ExCAPE-ML (official gene symbols) and to the species present in ExCAPE-DB (i.e. rat, mouse and human). Duplicated compound-target pairs were aggregated using median pXC50 activity. We retrieved 3 million AstraZeneca data points documenting 854,171 compounds and 20.6 million Janssen data points annotating 2,134,870 compounds. All compounds were standardized according to ExCAPE-DB’s standardization protocol. Compounds present in the ExCAPE-ML dataset were discarded from the AstraZeneca and Janssen datasets. This filtering step resulted in an AstraZeneca dataset of 808,699 molecules covering 352 targets out of the 526 targets of ExCAPE-ML. The same step resulted in a Janssen dataset of 1,794,089 compounds covering 465 targets with a total of 19 million data points. An overview about the compound distributions across the targets for the AstraZeneca dataset and the Janssen dataset is visualized by Fig. 1.

For the discarded compounds from the AstraZeneca and the Janssen datasets (that overlap with ExCAPE-ML) we computed a contingency table (see Table 1) between ExCAPE-ML and the respective company target-compound labels. The correlation between these labels is high, with a Matthew’s correlation coefficient of 0.74 and 0.91 for AstraZeneca and Janssen datasets, respectively. About 90% of the target-compound labels are identical underpinning the idea that models trained on public data can indeed be used to inform drug discovery efforts in companies.

**Table 1:** Contingency tables ExCAPE-ML vs. company datasets

		AstraZeneca			Janssen		
		Active	Inactive	Sum	Active	Inactive	Sum
ExCAPE-ML	Active	0.598	0.043	0.64	0.422	0.030	0.45
	Inactive	0.077	0.283	0.36	0.013	0.535	0.55
	Sum	0.67	0.33	1.00	0.44	0.56	1.00

Contingency tables for labels of ExCAPE-ML compounds being available also in the company datasets. The values are relative frequencies of the number of target-compound labels being characterized as active/inactive by ExCAPE-ML and the respective company dataset

### Descriptors

In this study, we used ECFP [38] descriptors (radius = 3, count values, unfolded) to represent compounds. In total, the molecular descriptor vectors contained 1,459,681 features to describe the complete ExCAPE-ML dataset. The descriptors were generated from isomeric SMILES by using AMBIT toolkit [39] which was developed based on the CDK toolkit [40].

### Prospective and retrospective model evaluation

As mentioned above, the main goal of this study was to evaluate the predictive classification performance of machine learning models, which were learned on publicly available data, on industrial data. This is referred to as prospective [41] model evaluation. Additionally, we evaluated the classification performance of machine learning models also on public data itself, which we refer to as retrospective model evaluation.

In contrast to prospective model evaluation, retrospective model evaluation requires a cross validation procedure. Since we also needed to determine hyperparameters for the considered machine learning algorithms and we wanted to avoid a hyperparameter selection bias (consider that the best hyperparameter combination might be dataset dependent [42]), which would arise when test set data leak into the hyperparameter choice, an additional cross validation step for hyperparameter selection as explained in Mayr et al. [29] was needed. This led to a nested cross validation procedure for retrospective model evaluation.

**Fig. 2:**
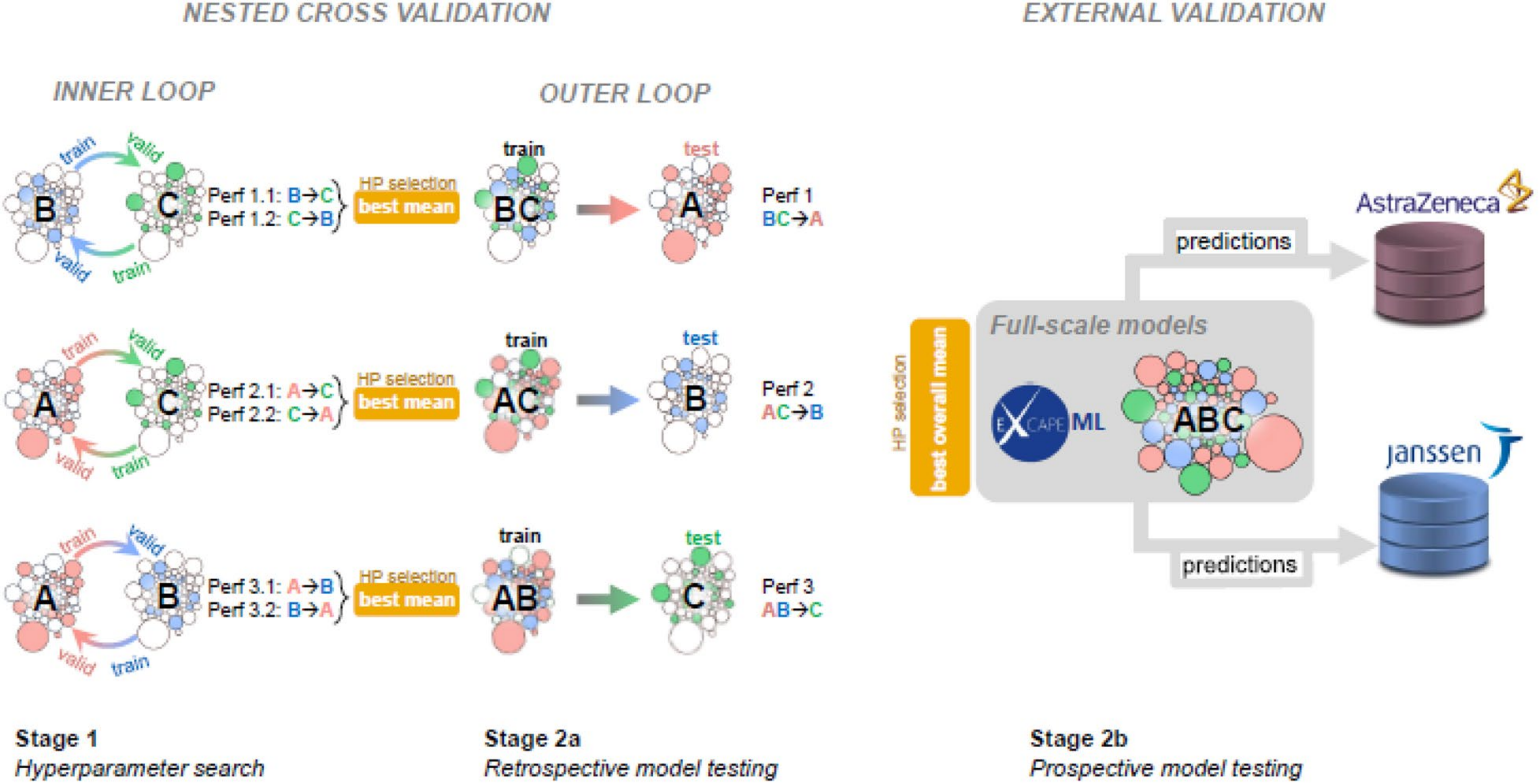
Prospective and Retrospective Model Evaluation with three folds (A, B, C). White and colored circles in the Figure represent clusters of compounds, where the size of the circles indicates the cluster sizes (nr. of compounds in the clusters). Colors indicate folds, to which clusters are assigned to, where white circles indicate folds, which are not used for building or evaluating a particular model. In stage 1, the inner loop, one of the three folds serves as the training set, one serves as a test set and the third one is kept aside as a test set for Stage 2a, the outer loop. The respective inner folds used in Stage 1 are merged to training sets for Stage 2a, the retrospective model testing stage. All folds together are merged to the training set for obtaining full-scale models in Stage 2b, the prospective model testing stage. Stage 1 is used for hyperparameter selection of Stage 2a and hyperparameter selection of Stage 2b. For retrospective model testing (Stage 2a) the two respective performance values (Perf X.Y) are averaged in each outer loop iteration step and the hyperparameter setting with the best ROC-AUC value is used for training models in Stage 2a, which finally gives performance values (Perf X) for retrospective model testing. For prospective model testing (Stage 2b) all six performance values (Perf X.Y) of the inner loop are averaged for hyperparameter selection. A final trained model on all data is then evaluated on AstraZeneca and Janssen industrial datasets

The whole process of hyperparameter selection as well as training and testing of machine learning models for prospective and retrospective evaluation is summarized in Fig. 2: The first two stages (Stage 1, Stage 2a) are the inner and outer loop components of a three-fold nested cross validation loop and are used to perform a retrospective performance evaluation [29, 43]. The third stage (Stage 2b) is prospective model evaluation, which is done with respect to ground truth from the two industry datasets.

### Evaluation of model performance

Our main prediction performance measure was the area under the receiver operating characteristic curve (ROC-AUC) metrics, which reflects the model’s ability to rank correctly active compounds higher than inactive compounds and which is an established performance measure for classification tasks. Additionally, we provide classification performance estimates by the Cohen-kappa score [44, 45] (Kappa) and the F1 score [46] (harmonic mean of precision and recall). Since we did not optimize thresholds for the respective models with respect to Kappa or F1, it is important to mention that our results should not be interpreted to provide a direct comparison of Kappa or F1 for the different methods. The results serve more as side-information to have a baseline for classification performance with respect to the native usage of the prediction methods.

### Hyperparameter selection

Our selection criterion for preferring one hyperparameter setting over another was based on the ROC-AUC scores of the individual models trained with a certain hyperparameter.

In the inner loop of the previously mentioned three-fold nested cross validation procedure, we trained a model on one data fold and finally estimated its ROC-AUC value on a second fold while skipping the remaining third fold (see Fig. 2). This process yielded in two performance estimates per skipped fold, i.e. we obtained six performance estimates per hyperparameter (Perf X.Y in Fig. 2) in Stage 1. From there on, we followed two separate scenarios: retrospective evaluation (Stage 2a) and prospective evaluation (Stage 2b).

For retrospective evaluation, we averaged each pair of performance estimates per skipped fold, hence resulting in three estimates. These estimates were then used to select the hyperparameter settings employed in each of the three outer cross validation steps respectively (Stage 2a). For prospective evaluation, we used the average performance estimate obtained over all the six estimates to select the hyperparameter setting for training a final model to be applied on the industrial datasets (Stage 2b).

It is worthwhile to note that for gradient boosting, which works in a single task scenario, hyperparameter settings were selected for each target individually, whereas this was not the case in the multitask scenario, i.e. deep learning and matrix factorization, for simplicity. Therefore, for the multitask case, we computed averages over the targets for hyperparameter selection.

### Definition of folds for retrospective model evaluation

In order to avoid overoptimistic performance estimation, i.e. avoid the compound series bias as described in Mayr et al. [29], ExCAPE-ML was split into three folds based on a chemical structure clustering [47] which should help assigning molecules from the same chemical series either to the training or to the test set, but not to both sets (Additional file 1: Notes S2).

### Used machine learning method implementations and their hyperparameters

For deep learning, we considered (standard) feed-forward fully connected deep neural networks (DNNs). We did not take into account neural graph convolution-based or neural sequence-based deep learning approaches, since the necessary network architecture design (hyperparameter) searches may have become very costly from a computational point of view and the question which deep learning architectures work best was not the main focus of this study. The hyperparameter search for our considered DNNs included up to 4 layers and up to 4096 hidden units per layer. We also applied the hyperparameter search on the overall architecture (ReLU [48, 49] architecture, SELU/SNN [50] architecture). The whole list of searched hyperparameters is listed in Additional file 1: Table S1. The number of input features was reduced by an upstream feature selection process, such that only features that exceeded a minimum occurrence threshold (more than 0.25% none-sparse entries) across the compounds in the respective training sets were kept. This resulted in between about 2300 and 2600 features (dependent on the respective training set from which a model is obtained). We applied dropout for regularization at the hidden units and used a sigmoid output layer matching the number of targets to predict (526 output nodes). The objective for training the networks was the minimization of the summed cross-entropies across the different targets, where targets, for which no training data were available, have been excluded from the summation (for details see Additional file 1: Notes S3). The networks were trained by a stochastic gradient descent (SGD) optimizer. For measuring classification performance by Kappa and F1, we used a threshold of 0.5 on the sigmoid output layer.

For matrix factorization (MF) the algorithm Macau [51] was used. Macau was implemented by the SMURFF [52] software package. Hyperparameters were tuned via grid-search, by varying the values of the dimension of the latent space systematically, the precision of the observations, the precision of the compound features and the number of samples collected from Gibbs sampling (see Additional file 1: Table S1). In practice, the total number of Gibbs samples was always set to 2000 but we varied the number of burn-in iterations by steps of 100 between 200 and 1800, thus effectively resulting in 17 different number-of-sample values ranging from 200 to 1800. For classification predictions, the predicted continuous activity values were considered as active if the predicted activity value was higher than or equal to 6 and inactive if it was lower; the final activity prediction was calculated by averaging across per-sample predictions.

Gradient Boosting models were built using XGBoost [53] (XGB). Hyperparameter tuning was performed by hyperparameter grid search exploring the value list in Additional file 1: Table S1. For computing Kappa and F1, we used the classification thresholds provided by the used python package.

It should be mentioned, that for XGB the classifiers for compound activity were trained independently for each target, which is in contrast to the training of target classifiers obtained by DNNs and MF.

For XGB and MF, the number of input features was trimmed down to a fixed set of 29,413 features by removing any feature with low variance (threshold variance is 0.05, which was applied to non-zero entries only). This set of features was used for all experiments with XGB and MF. It should be noted, that the number of input features for DNNs was smaller (although being selected from the same original set of features as the selected 29,413 features) in order to allow training more DNNs in parallel for hyperparameter search on a GPU, which would otherwise not have been possible because of GPU memory restrictions.

## Results and discussion

### Retrospective validation performance

**Fig. 3:**
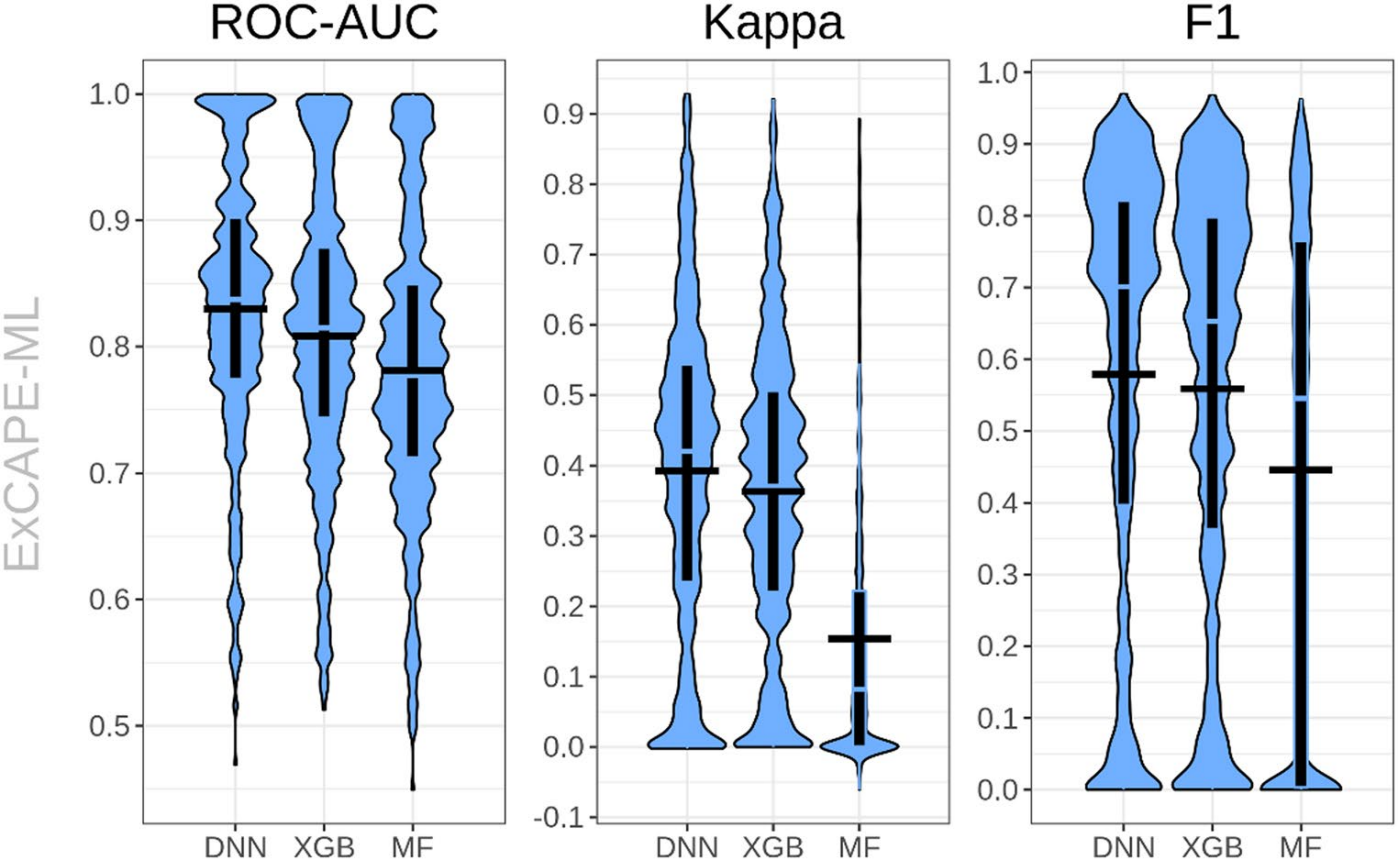
ROC-AUC, Kappa and F1-score performances of DNN, XGB and MF models on the ExCAPE-ML dataset. Violin plots illustrate the distribution of individual target performances, boxplots represent the interquartile range, with median value in transparent and average as the horizontal black segment

In the retrospective part of this study, we estimated the predictive classification performance of machine learning methods on ExCAPE-ML itself by cross validation. Table 2 shows mean cross validation model performance values (ROC-AUC, Kappa, F1) together with their standard deviations for test folds of the outer loop from the retrospective evaluation procedure over the individual targets. For the errors, thereby, first cross validation performances per target are computed, and then standard deviations over the individual targets. Additionally, Fig. 3 shows violin plots of the cross validation performances. Especially, ROC-AUC statistics suggests, that DNNs outperform XGB and MF with p-values of 8.01e-48 and 1.80e-71 for the alternative hypothesis that DNNs have a better mean ROC-AUC than XGB or MF, respectively (paired Wilcoxon test).

**Table 2:** Retrospective evaluation performance

	Metric			Null Hyp.: DNN AUC < row AUC	
Algorithm	ROC-AUC	Kappa	F1	Wilcoxon test	Sign test
DNN	0.83 ± 0.11	0.39 ± 0.23	0.58 ± 0.30		
XGB	0.81 ± 0.11	0.36 ± 0.21	0.56 ± 0.30	8.01e−48	7.90e−50
MF	0.78 ± 0.11	0.15 ± 0.20	0.45 ± 0.34	1.80e−71	1.14e−84

Retrospective evaluation performance values (mean and standard deviation across targets) for the considered machine learning algorithms together with p-values of tests comparing the ROC-AUC of the respective algorithm in the table row with that of DNNs

The retrospective analysis on ExCAPE-ML is in concordance with other comparative studies [29], which indicate that deep learning is a proficient method for drug target prediction

Furthermore, all three considered methods have been shown to work on to a certain extent on previously unseen compound series. This suggests the capability of the three methods to learn predictive models for the activities of compound series not present in the training set and in turn highlights that the knowledge learnt from ExCAPE-ML has the potential to be transferred to external datasets. In order to investigate this further, and to understand better how well models trained with ExCAPE-ML data can predict external data, we performed a prospective evaluation by applying ExCAPE-ML models, trained on all ExCAPE-ML data, to two external industrial datasets.

### Prospective validation performance

**Fig. 4:**
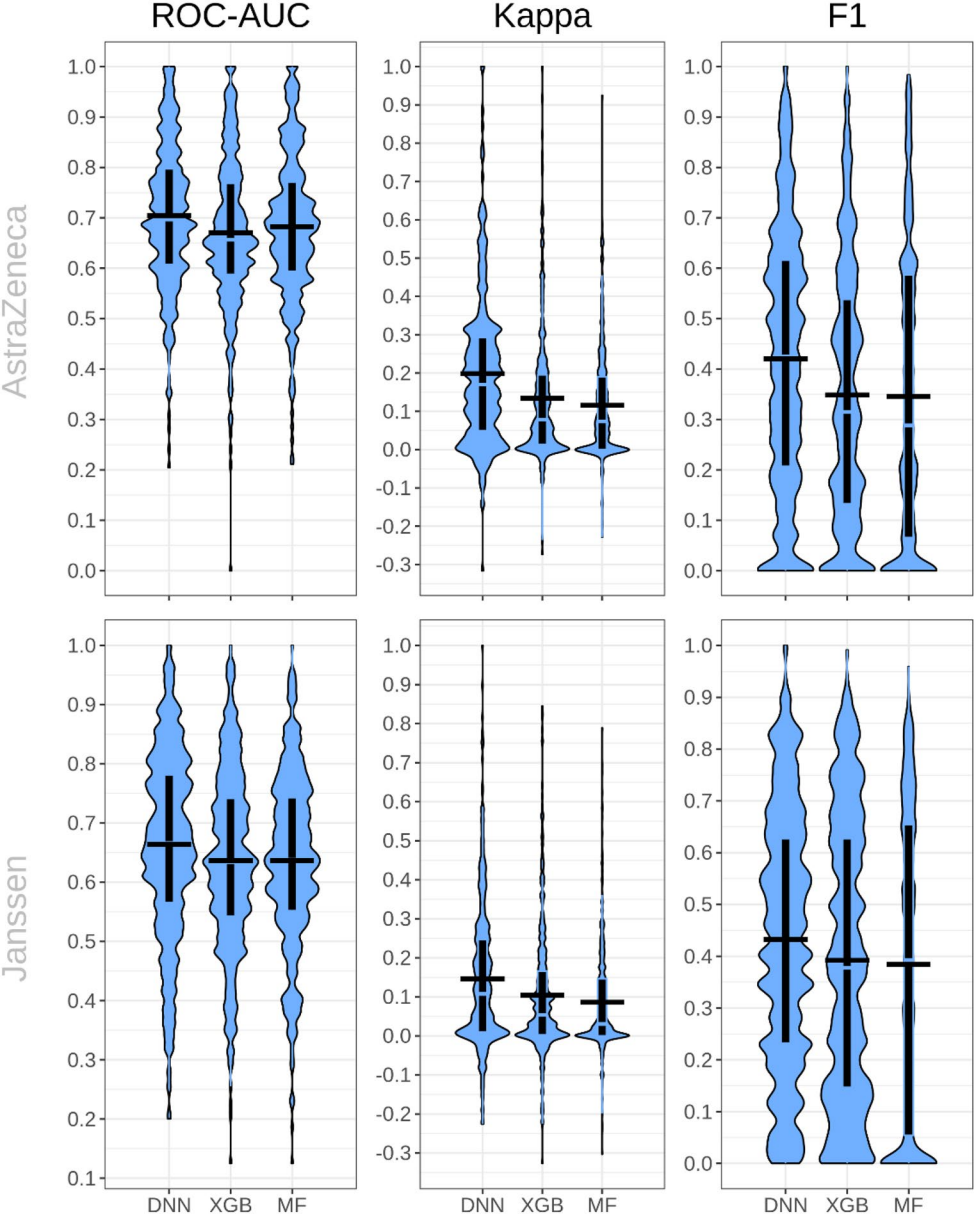
ROC-AUC, Kappa and F1-score performances of DNN, XGB and MF models on AstraZeneca and Janssen datasets. Violin plots illustrate the distribution of individual target performances, boxplots represent the interquartile range, with median value in transparent and average as the horizontal black segment

In the prospective part of this study, we assessed how well machine learning models trained on public data can be transferred to internal pharmaceutical industry data by applying them to two industrial, in-house datasets. The classification performance statistics (ROC-AUC, Kappa, F1) for prospective validation of our three prediction models on the AstraZeneca and Janssen datasets is given by Table 3 and further visualized by Fig. 4.

**Table 3:** Prospective evaluation performance

Algorithm	Metric	AstraZeneca	Janssen
DNN	ROC-AUC	0.70 ± 0.14	0.66 ± 0.16
	Kappa	0.20 ± 0.19	0.15 ± 0.19
	F1	0.42 ± 0.26	0.43 ± 0.24
XGB	ROC-AUC	0.67 ± 0.15	0.64 ± 0.15
	Kappa	0.13 ± 0.17	0.10 ± 0.17
	F1	0.35 ± 0.25	0.39 ± 0.27
MF	ROC-AUC	0.68 ± 0.15	0.64 ± 0.15
	Kappa	0.12 ± 0.15	0.09 ± 0.14
	F1	0.35 ± 0.29	0.38 ± 0.30

Prospective evaluation performance values (mean and standard deviation across targets) for the considered machine learning algorithms

In general, ROC-AUC prediction performance on industrial datasets is moderate compared to the estimations made in the retrospective analysis (13 to 17% lower for the AstraZeneca dataset and 18 to 21% lower for the Janssen dataset). Nevertheless, despite predicting unseen molecules, the predictions for at least 25% of the targets reached a ROC-AUC ≥ 0.7 with all three methods simultaneously (99 out of 352 targets for the AstraZeneca dataset and 116 out of 465 targets for the Janssen dataset), hence demonstrating that the public models are still valuable for prospective predictions.

A Wilcoxon test could reject the hypothesis that the mean ROC-AUC of DNNs is below 0.6 with a p-value of 7.4e−31 for the AstraZeneca dataset and a p-value of 3.3e−17 for the Janssen dataset. For the other algorithms, we obtained also highly significant p-values, rejecting the hypothesis that prediction scores were random. Similarly, for targets in the AstraZeneca dataset and targets in the Janssen dataset, all methods exhibit Kappa scores, for which the null hypothesis, that Kappa is negative, could be rejected (e.g. for DNNs 7.2e−51 for the AstraZeneca dataset and 2.4e−51 for the Janssen dataset). This is especially notable, since the classification thresholds were not explicitly optimized for obtaining good Kappa scores.

A reason for the performance decrease compared to ExCAPE-ML could be that ExCAPE-ML has a different compound activity distribution than the AstraZeneca and Janssen datasets, which can be seen, when Fig. 1 is compared to Additional file 1: Fig. S1. On ExCAPE-ML there is a large number of targets that presumably arise from high-throughput screening and where the activity ratio is close to zero. This activity distribution property cannot be observed in the considered industrial datasets. Further, other, at least equally important, but harder quantifiable reasons for the performance decrease might be different assay technologies or a different chemical space of compounds, which is not reflected by the public dataset and which makes accurate predictions difficult.

If we compare the considered machine learning methods to each other, a consistent result to the retrospective performance analysis is that DNNs outperform XGB and MF. This is especially remarkable given the sizes of company-internal datasets and the differences in activity distributions. The respective p-values of Wilcoxon signed rank tests and sign tests are given by Table 4. It should be mentioned that the method comparison was not the main focus here and since the absolute performance differences were moderate, possibly further investigations will be needed, to better understand under which conditions a method excels another one.

**Table 4:** Prospective Performance Comparison

	Null Hyp.: DNN AUC < row AUC			
	Wilcoxon test		Sign test	
Algorithm	AstraZeneca	Janssen	AstraZeneca	Janssen
XGB	7.17e−15	2.62e−15	1.27e−14	6.84e−14
MF	4.01e−09	3.23e−11	2.98e−08	3.04e−11

p-values of comparing the respective algorithm prospective ROC-AUC evaluation performance in the table row with that of DNNs using two different statistical tests

We further investigated whether there would be a dependency of the prediction performance on the industrial datasets from the available training set size of the target in ExCAPE-ML. We found the Spearman correlation between training set sizes and the DNN ROC-AUC values over the targets to be 0.18 and 0.09 for the AstraZeneca and the Janssen dataset respectively. A correlation test showed significance at a threshold of 0.01. Additional file 1: Fig. S3 underpins the result, that there might be a slight correlation, however it isn’t large.

Overall, we can conclude (1) that for a big number of targets successful model transfer is observed with a certain performance decrease and (2) Deep Learning outperforms methods investigated in this classification study on industrial data in which aggregated ranking performance metrics across several diverse targets were used as the main criterion.

### Prediction performances of different target families

**Fig. 5:**
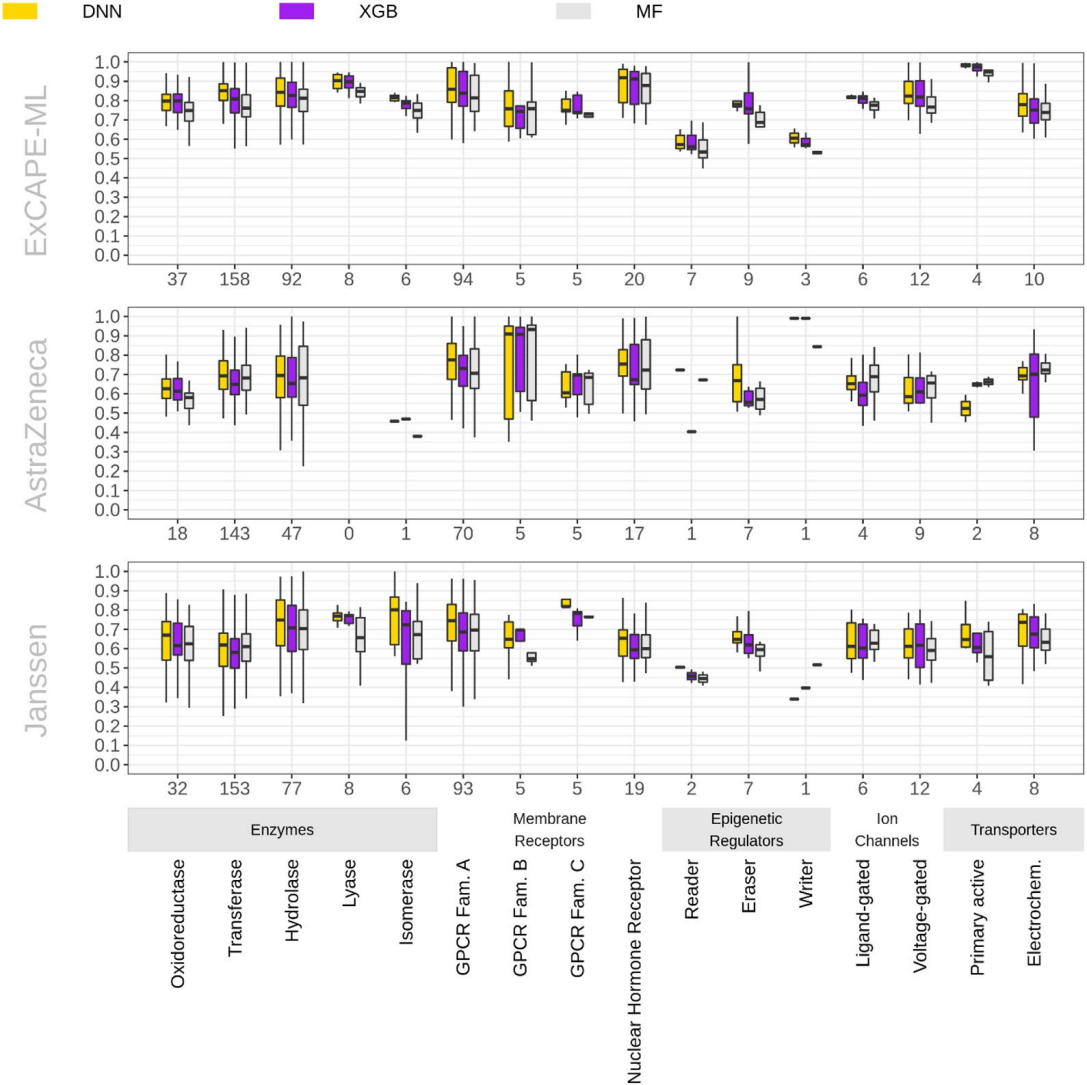
Target family breakdown for ExCAPE-ML, AstraZeneca and Janssen predictions. The numbers on the horizontal axis represent the number of targets corresponding to the target family and dataset. The vertical axis represents the AUC value

In the final part of this study, we investigated performances obtained for various types of biological activities. In order to do so, we analyzed the distribution of ROC-AUC values across different protein target families (see Fig. 5). The first main column of Fig. 5 shows the prediction performances across target families for retrospective model evaluation. It can be observed, that machine learning algorithms seem to work well for predicting diverse types of biological activities. This is a similar observation to that found for assays in the study of Mayr et al. [29]. The second and third main columns of Fig. 5 show target family performances for the AstraZeneca and the Janssen datasets respectively. Although we observe that there is no perfect correlation between family performances found on ExCAPE-ML and the respective performances on the AstraZeneca and Janssen datasets, model transfer works similarly well across such diverse targets as membrane receptors, ion channels or enzymes catalyzing metabolic reactions.

Further, we investigated for each target, which of the three machine learning methods worked best and checked for each target family whether the winning probability of DNNs is less than a third or greater. The p-value results are given in Table 5 together with the numbers of targets won by DNNs and the overall numbers of targets for each target family. In general, we conclude, that for large enough target families, we can reject the null hypothesis that the winning probability of DNNs is only a third on the public dataset as well as on the two industrial datasets, indicating that Deep Learning is advantageous for most of the target families. Noticeable exceptions from this conclusion seem to be the Transferase family of the Janssen dataset, where MF wins 65 targets or the Oxidoreductase family of the AstraZeneca dataset, where XGB is the best method for 9 targets.

**Table 5:** Target Family Performance Comparison

	ExCAPE-ML		AstraZeneca		Janssen	
Target family	Targets w./sz.	p-value	Target w./sz.	p-value	Targets w./sz.	p-value
Oxidoreductase	25/37	*2.17e*−*05*	7/18	3.91e−01	16/32	3.77e−02
Transferase	*141/158*	*1.58e*−*48*	*77/143*	*3.64e−07*	*60/153*	*7.38e−02*
Hydrolase	63/92	*6.24e*−*12*	21/47	6.96e−02	36/77	*9.91e*−*03*
Lyase	4/8	2.59e−01	0/0		4/8	2.59e−01
Isomerase	4/6	1.00e−01	0/1	1.00e + 00	4/6	1.00e−01
GPCR Fam. A	76/94	*3.78e*−*21*	34/70	*5.87e*−*03*	66/93	*1.39e*−*13*
GPCR Fam. B	3/5	2.10e−01	0/5	1.00e + 00	2/5	5.39e-01
GPCR Fam. C	3/5	2.10e−01	1/5	8.68e-01	5/5	*4.12e-03*
Nuclear Hormone Receptor	14/20	*8.79e*−*04*	9/17	7.55e−02	13/19	*1.87e*−*03*
Reader	2/7	7.37e−01	1/1	3.33e−01	2/2	1.11e−01
Eraser	7/9	*8.28e−03*	5/7	4.53e-02	4/7	1.73e-01
Writer	3/3	3.70e−02	1/1	3.33e−01	0/1	1.00e + 00
Ligand-gated	3/6	3.20e−01	1/4	8.02e−01	2/6	6.49e−01
Voltage-gated	6/12	1.78e−01	3/9	6.23e−01	6/12	1.78e−01
Primary active	3/4	1.11e−01	0/2	1.00e + 00	2/4	4.07e−01
Electrochem.	7/10	1.97e−02	2/8	8.05e−01	3/8	5.32e−01
Overall	364/476	*1.26e*−*82*	162/338	*2.02e*−*08*	225/438	*5.95e*−*15*

Number of targets won (w.) by DNNs from a target family, size of target family (sz.) and p-values of binomial tests for each target family class, with the null hypothesis that the probability of being the best method for a certain target is less than 1/3 for DNNs when compared to XGB and MF

p-values below the significance threshold of 0.01 are in italics

## Conclusions

In this study, we utilized public bioactivity data to derive predictive machine learning models for classification, with the goal to transfer them to industrial data and evaluate their performances there. In a retrospective analysis we assessed the performance of our machine learning models on public data. We confirmed previous observations that multitask Deep Learning outperforms other state-of-the-art target prediction methods. In a prospective study, we directly transferred our learned models to industrial datasets and evaluated the predictive quality on those molecules. There our most important observation was, that particular models can still preserve good predictive quality with ROC-AUC performances, which were on average between about 0.65 and 0.70. Although the performance decreased, there are nevertheless a lot of useful models for specific targets with an AUC of at least 0.70 on both industrial datasets. Furthermore, successful model transfer works across different target families. We could finally observe that Deep Learning derived target prediction models are in this study superior to models derived by other machine learning algorithms also on industrial datasets.

We think the results of our study are interesting for both, drug discovery and machine learning. As mentioned, data distributions between public databases and industry databases might be different; it is therefore notable, that prediction performances transfer well to in-house data of companies. From a machine learning point of view, this study might serve as a proof-of-concept for successful model transfer.

## Supplementary information

**Additional file 1: Fig. S1.**  Compound distributions across the targets for the ExCAPE-ML dataset. **Fig. S2.**  Targets per protein families. **Notes S1.** ExCAPE-ML Target Families. **Notes S2.** Clustering and Assignment of Clusters to Folds. **Notes S3.** Objective Function for Deep Learning. **Table S1.** Considered machine learning algorithm hyperparameters. **Fig. S3.**  DNN ROC-AUC performances on industrial datasets vs. Training set size.

## Data Availability

The ExCAPE-ML dataset, on which the retrospective analysis is applied and on which the models for the prospective analysis are learnt, is publicly available at https://zenodo.org/record/3559987 The industrial datasets from AstraZeneca and Janssen, cannot be provided to the public. Code, used for this study, is available at https://github.com/ExCAPE-Project.

## References

[1] Ekins S, Puhl AC, Zorn KM (2019). Exploiting machine learning for end-to-end drug discovery and development. Nat Mater.

[2] Vamathevan J, Clark D, Czodrowski P (2019). Applications of machine learning in drug discovery and development. Nat Rev Drug Discovery.

[3] Wang L, Ding J, Pan L (2019). Artificial intelligence facilitates drug design in the big data era. Chemometrics Intell Lab Syst.

[4] Gaulton A, Hersey A, Nowotka M (2017). The ChEMBL database in 2017. Nucleic Acids Res.

[5] Kim S, Chen J, Cheng T (2019). PubChem 2019 update: improved access to chemical data. Nucleic Acids Res.

[6] Lavecchia A (2015). Machine-learning approaches in drug discovery: methods and applications. Drug Discov Today.

[7] Bender A, Glen RC (2004). Molecular similarity: a key technique in molecular informatics. Org Biomol Chem.

[8] Martínez-Jiménez F, Papadatos G, Yang L (2013). Target prediction for an open access set of compounds active against *Mycobacterium tuberculosis*. PLoS Comput Biol.

[9] Koutsoukas Alexios, Simms Benjamin, Kirchmair Johannes (2011). From in silico target prediction to multi-target drug design: current databases, methods and applications. J Proteomics.

[10] Bosc N, Atkinson F, Felix E (2019). Large scale comparison of QSAR and conformal prediction methods and their applications in drug discovery. J Cheminform.

[11] Breiman L (2001). Random Forests. Mach Learn.

[12] Cortes C, Vapnik V (1995). Support-vector networks. Mach Learn.

[13] Sydow D, Burggraaff L, Szengel A (2019). Advances and challenges in computational target prediction. J Chem Inf Model.

[14] Ricci F, Rokach L, Shapira B, Ricci F (2011). Introduction to recommender systems handbook. Recommender systems handbook.

[15] Deng L, Hinton G, Kingsbury B (2013). New types of deep neural network learning for speech recognition and related applications: an overview. IEEE International Conference on Acoustics, Speech and Signal Processing (ICASSP), 2013: 26-31 May 2013, Vancouver Convention Center, Vancouver, British Columbia.

[16] Krizhevsky A, Sutskever I, Hinton GE, Pereira F, Burges CJC, Bottou L (2012). ImageNet classification with deep convolutional neural networks. Advances in neural information processing systems 25.

[17] Simonyan K, Zisserman A (2014) Very deep convolutional networks for large-scale image recognition. International Conference on Learning Representations (ICLR) 2015. arXiv:1409.1556

[18] He K, Zhang X, Ren S et al. (2016) Deep residual learning for image recognition. In: The IEEE Conference on computer vision and pattern recognition (CVPR)

[19] Xie J, Liu R, Luttrell J (2019). Deep learning based analysis of histopathological images of breast cancer. Front Gene.

[20] Collobert R, Weston J (2008) A unified architecture for natural language processing. In: McCallum AK, Roweis S (eds) Proceedings, Twenty-fifth International Conference on machine learning: [Helsinki, Finland, 5–9 July, 2008]. University of Helsinki, Helsinki, Finland, pp 160–167

[21] Goodfellow I, Pouget-Abadie J, Mirza M et al. (2014) Generative adversarial nets. In: Ghahramani Z, Welling M, Cortes C, et al. (eds) Advances in neural information processing systems 27. Curran Associates, Inc, New York, pp 2672–2680

[22] Chen H, Engkvist O, Wang Y (2018). The rise of deep learning in drug discovery. Drug Discov Today.

[23] Wenzel J, Matter H, Schmidt F (2019). Predictive multitask deep neural network models for ADME-Tox properties: learning from large data sets. J Chem Inf Model.

[24] Ma J, Sheridan RP, Liaw A (2015). Deep neural nets as a method for quantitative structure-activity relationships. J Chem Inf Model.

[25] Ramsundar B, Liu B, Wu Z (2017). Is multitask deep learning practical for pharma?. J Chem Inf Model.

[26] Xu Y, Ma J, Liaw A (2017). Demystifying multitask deep neural networks for quantitative structure-activity relationships. J Chem Inf Model.

[27] Dahl GE, Jaitly N, Salakhutdinov R (2014) Multi-task Neural Networks for QSAR Predictions. arXiv:1406.1231

[28] Mayr A, Klambauer G, Unterthiner T (2016). DeepTox: toxicity prediction using deep learning. Front Environ Sci..

[29] Mayr A, Klambauer G, Unterthiner T (2018). Large-scale comparison of machine learning methods for drug target prediction on ChEMBL. Chem Sci.

[30] Sun J, Jeliazkova N, Chupakhin V (2017). ExCAPE-DB: an integrated large scale dataset facilitating Big Data analysis in chemogenomics. J Cheminform.

[31] Koutsoukas A, Lowe R, Kalantarmotamedi Y (2013). In silico target predictions: defining a benchmarking data set and comparison of performance of the multiclass Naïve Bayes and Parzen-Rosenblatt window. J Chem Inf Model.

[32] Mervin LH, Afzal AM, Drakakis G (2015). Target prediction utilising negative bioactivity data covering large chemical space. J Cheminform.

[33] Kalliokoski T, Kramer C, Vulpetti A (2013). Comparability of mixed IC_50_ data—a statistical analysis. PLoS ONE.

[34] Hasselgren C, Muthas D, Ahlberg E, Bajorath J (2013). Chemoinformatics and beyond: moving from simple models to complex relationships in pharmaceutical computational toxicology. Chemoinform Drug Discov.

[35] van Vlijmen H, Desjarlais RL, Mirzadegan T (2017). Computational chemistry at Janssen. J Comput Aided Mol Des.

[36] Tipton KF (1994) Nomenclature Committee of the International Union of Biochemistry and Molecular Biology (NC-IUBMB). Enzyme nomenclature. Recommendations (1992). Supplement: corrections and additions. Eur J Biochem.

[37] Apweiler R, Bairoch A, Wu CH (2004). UniProt: the Universal Protein knowledgebase. Nucleic Acids Res.

[38] Rogers D, Hahn M (2010). Extended-connectivity fingerprints. J Chem Inf Model.

[39] Kochev NT, Paskaleva VH, Jeliazkova N (2013). Ambit-tautomer: an open source tool for tautomer generation. Mol Inform.

[40] Willighagen EL, Mayfield JW, Alvarsson J (2017). The Chemistry Development Kit (CDK) v2.0: atom typing, depiction, molecular formulas, and substructure searching. J Cheminform.

[41] Ekins S (2016). The next era: deep learning in pharmaceutical research. Pharm Res.

[42] Zhou Y, Cahya S, Combs SA (2019). Exploring tunable hyperparameters for deep neural networks with industrial ADME data sets. J Chem Inf Model.

[43] Baumann D, Baumann K (2014). Reliable estimation of prediction errors for QSAR models under model uncertainty using double cross-validation. J Cheminform.

[44] Cohen J (1960). A coefficient of agreement for nominal scales. Educ Psychol Measur.

[45] Artstein R, Poesio M (2008). Inter-coder agreement for computational linguistics. Comput Linguist.

[46] Powers DM (2011). Evaluation: from precision, recall and F-measure to ROC, informedness, markedness & correlation. J Mach Learn Technol.

[47] Butina D (1999). Unsupervised data base clustering based on daylight’s fingerprint and tanimoto similarity: a fast and automated way to cluster small and large data sets. J Chem Inf Comput Sci.

[48] Nair V, Hinton GE (2010) Rectified linear units improve restricted boltzmann machines. In: Proceedings of the 27th International Conference on machine learning. Omnipress, Aliso Viejo, pp 807–814

[49] Xavier Glorot, Antoine Bordes, Yoshua Bengio (2011) Deep Sparse Rectifier Neural Networks Intelligence and Statistics, AISTATS 2011, Fort Lauderdale, USA, April 11-13, 2011. In: Geoffrey J. Gordon, David B. Dunson, Miroslav Dudık (eds) Proceedings of the Fourteenth International Conference on artificial intelligence and statistics, AISTATS 2011, Fort Lauderdale, April 11–13, 2011. JMLR.org, pp 315–323

[50] Klambauer G, Unterthiner T, Mayr A, Guyon I, Luxburg UV, Bengio S (2017). Self-normalizing neural networks. Advances in neural information processing systems 30.

[51] Simm J, Arany A, Zakeri P et al. (2017) Macau: Scalable Bayesian factorization with high-dimensional side information using MCMC. In: 2017 IEEE 27th International Workshop on machine learning for signal processing (MLSP). IEEE, New York, pp 1–6

[52] Vander Aa T, Chakroun I, Ashby TJ et al. (2019) SMURFF: a high-performance framework for matrix factorization. arXiv:1904.02514

[53] Chen T, Guestrin C (2016) XGBoost: A scalable tree boosting system. In: Proceedings of the 22nd ACM SIGKDD International Conference on knowledge discovery and data mining. ACM, New York, pp 785–794

[54] Cima V, Böhm S, Martinovič J et al. (2018) HyperLoom. In: PARMA-DITAM 2018 proceedings: 9th Workshop on Parallel programming and run-time management techniques for Many-core Architectures; 7th Workshop on design tools and architectures for multicore embedded computing platforms. January 23, 2018, Manchester, United Kingdom. The Association for Computing Machinery, New York, pp 1–6

