# Supplementary material for: Industry-scale application and evaluation of deep learning for drug target prediction
Source: J Cheminform. 2020 Apr 19;12:26. doi: 10.1186/s13321-020-00428-5 (PMC7169028; doi:10.1186/s13321-020-00428-5)
Supplement: Supplementary file 1 — Additional file 1: Fig. S1. Compound distributions across the targets for the ExCAPE-ML dataset. Fig. S2. Targets per protein families. Notes S1. ExCAPE-ML Target Families. Notes S2. Clustering and Assignment of Clusters to Folds. Notes S3. Objective Function for Deep Learning. Table S1. Considered machine learning algorithm hyperparameters. Fig. S3. DNN ROC-AUC performances on industrial datasets vs. Training set size. [file 13321_2020_428_MOESM1_ESM.pdf]

Supplementary information:

Industry-scale Application and Evaluation of Deep Learning for Drug Target Prediction

Noé Sturm[#1], Andreas Mayr[#2], Thanh Le Van[3], Vladimir Chupakhin[4], Hugo Ceulemans[3], Joerg Wegner,[3] Jose-Felipe Golib-Dzib[5], Nina Jeliazkova[6], Yves Vandriessche[7], Stanislav Böhm[8], Vojtech Cima[8], Jan Martinovic[8], Nigel Greene[1], Tom Vander Aa[9], Thomas J. Ashby[9], Sepp Hochreiter[2], Ola Engkvist[10], Günter Klambauer[+2], Hongming Chen[+10]

1. Clinical Pharmacology and Safety Science, R&D BioPharmaceuticals, AstraZeneca, Pepparedsleden 1, 43183, Mölndal, Sweden.

2. LIT AI Lab & Institute for Machine Learning, Johannes Kepler University Linz, Altenberger Str. 69, 4040 Linz, Austria.

3. High-Dimensional Biology & Discovery Data Sciences, Discovery Sciences, Janssen Pharmaceutica, Turnhoutseweg 30, 2349, Beerse, Belgium.

4. High-Dimensional Biology & Discovery Data Sciences, Discovery Sciences, Janssen R&D, 1400 McKean Rd, 19002, Spring House, Pennsylvania US.

5. High-Dimensional Biology & Discovery Data Sciences, Discovery Sciences, Janssen Cilag SA, Calle Río Jarama, 75A, 45007, Toledo, Spain.

6. Ideaconsult Ltd., 4. Angel Kanchev Str., 1000 Sofia, Bulgaria.

7. Intel Corporation, Data Center Group, Veldkant 31, 2550 Kontich, Belgium.

8. IT4Innovations, VSB – Technical University of Ostrava, 17. Listopadu 2172/15, 70800, Ostrava-Poruba, Czech Republic.

9. Exascience Lab, Imec, Kapeldreef 75, B-3001 Leuven, Belgium.

10. Hit Discovery, Discovery Sciences, R&D BioPharmaceuticals, AstraZeneca, Pepparedsleden 1, 43183, Mölndal, Sweden.

# Joint first authors.

+ Joint last authors.

Corresponding authors:

Noé Sturm
noesturm@gmail.com

Günter Klambauer
klambauer@ml.jku.at

Chen Hongming
Hongming.Chen71@hotmail.com

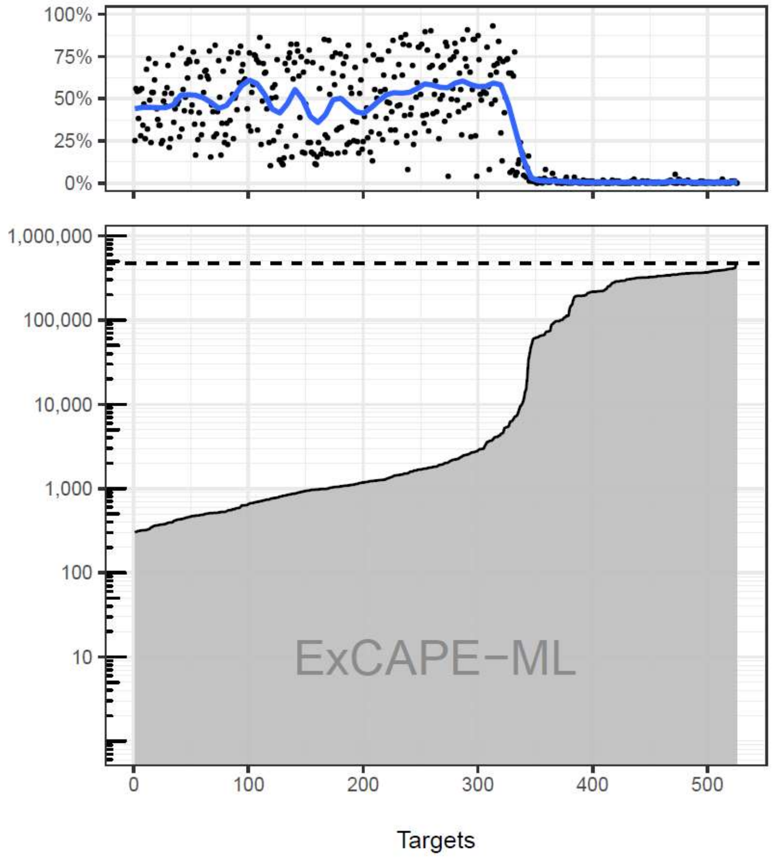

Fig. S1 Compound distributions across the targets for the ExCAPE-ML dataset. In the lower panel, the y-axis shows the number of compounds for targets represented by the x-axis, where the targets are sorted according to the number of compounds. The horizontal dashed line represents the maximum number of compounds per target observed in the datasets. In the upper panel, a point represents the activity ratio of a target; targets are sorted the same way as in the lower panel. The curve in the upper panel is a smooth average.

Notes S1: ExCAPE-ML Target Families

We categorized the targets of ExCAPE-ML according to the ChEMBL protein hierarchy, which resulted in six target classes including a class of miscellaneous targets (see Figure S2). The IUPAC enzyme nomenclature was chosen for characterizing enzymes. All the enzyme's EC numbers were fetched from UniProt after mapping Entrez gene identifiers to reviewed UniProt accession codes.

Enzymes are the most represented protein family with 301 targets representing five classes of enzymes: transferases (158 out of which 149 are kinases), hydrolases (92), oxidoreductases (37), lyases (8) and isomerases (6). Membrane receptors are the second most represented target family with 107 targets, mainly representing GPCR family A (94) but also GPCR families B (5) and C (5), and 3 unclassified membrane receptors. Transcription factors are the third most represented target family with 24 proteins describing nuclear hormone receptors from subfamily 1 (12 targets), subfamily 3 (six targets), subfamilies 5 and 2 (one target each) and 4 unclassified targets. The ion channel proteins category includes mainly voltage-gated (12) and ligand-gated (6) ion channels, with four other ion channels. The remaining 72 targets are classified as miscellaneous. The complete target annotation file including target symbols, target Entrez gene identifiers, UniProt accession codes, EC numbers, protein full names and ChEMBL protein classes is available at https://zenodo.org/deposit/3239499.

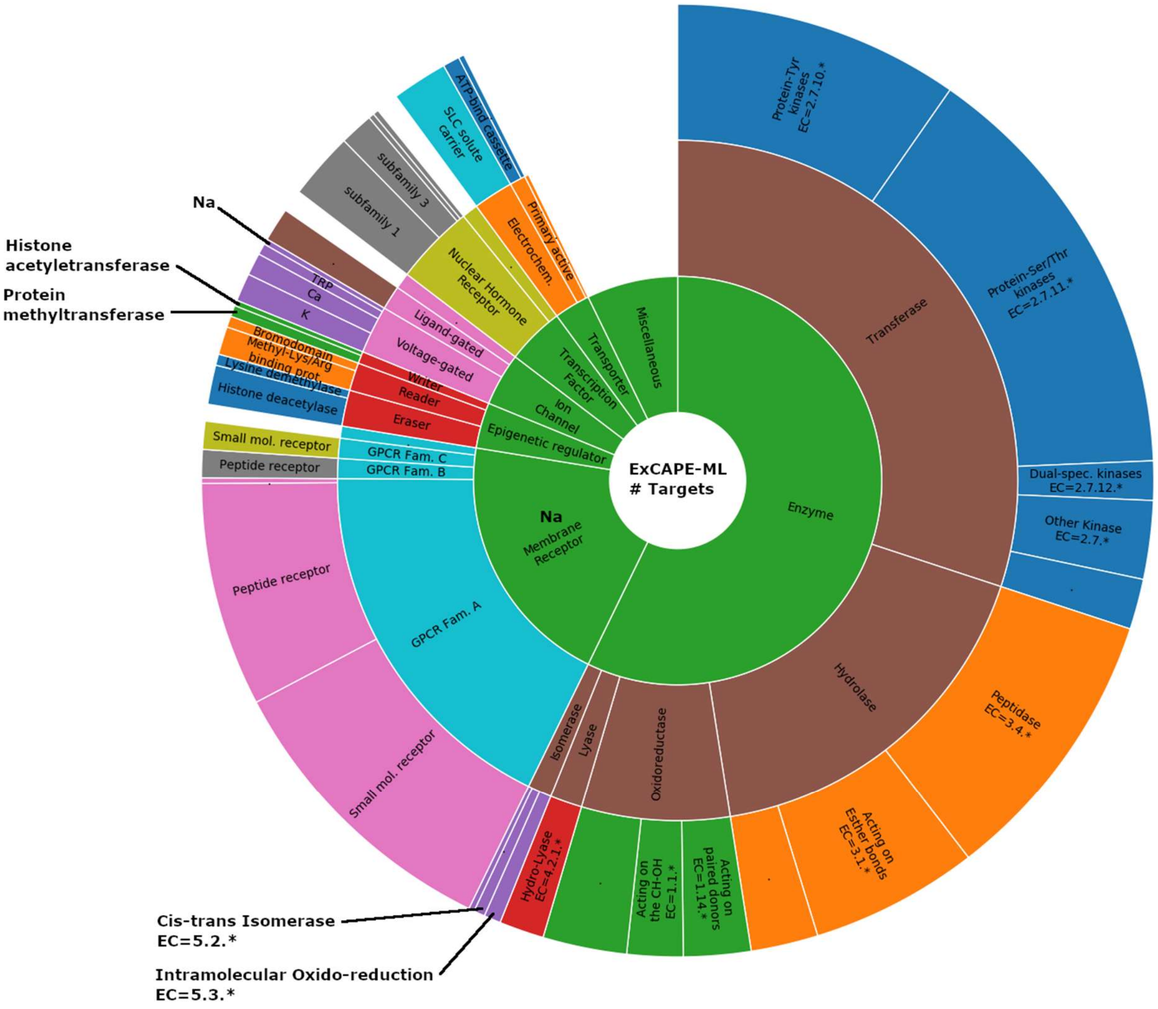

Fig. S2 Targets per protein families. Size of the boxes are proportional to the number of targets within each category. Boxes without the "dot" label represent proteins from this family unclassified in this plot. Enzyme class names are based on NC-IUBMB. Other protein families are based on ChEMBL protein classification.

Notes S2: Clustering and Assignment of Clusters to Folds

The clustering was performed by a sphere exclusion clustering algorithm (similarity threshold of 0.45 based on ECFP descriptors). All clusters were then shuffled regardless of their size and evenly distributed in three folds based on their new order. Due to the equivalent average sizes of the clusters, the three folds contained similar amounts of compounds. This random shuffling process was repeated until a thousand different folding solutions were found. Lastly, for each valid folding solution, three different configurations of training and test sets were inspected to get the list of unique targets overlapping between training and test sets. The folding solution with the largest overlapping target list was selected as the final folding solution, it contained 526 targets for around 315,000 compounds in each fold.

Notes S3: Objective Function for Deep Learning

For deep neural networks, we used a sum of cross entropy terms across different targets as the objective function for the classification problem, we considered: In equation (1) DNN denotes the sigmoid target prediction outputs (being between 0 and 1) of a deep neural network, t denotes a target (i.e. one of the sigmoid outputs), x describes a sample (compound), which is represented by its features, y_t denotes the (true) label of a sample (being either 0 or 1), and mask_t denotes, whether training label data is available or not for the current sample and target t (represented by 1 if target data is available and 0 if no target data is available).

$$-\sum_{t=1}^{T} \mathrm{mask}_t\left(y_t \log\left(\mathrm{DNN}_t(x)\right) + (1 - y_t)\log\left(1 - \mathrm{DNN}_t(x)\right)\right) \quad (1)$$

It should be noted, that in the case, no training label data is available (i.e. $\mathrm{mask}_t = 0$), the value y_t might be arbitrary, as it does not affect the value of the whole sum in this case.

	Parameters	Considered values
DNN	Architecture	1024×1024×1024
		2048×2048×2048
		4096×4096×4096
		2048×2048
		2048×2048×2048×2048
	Learning Rate	0.1, 0.01
	Input dropout	0.0, 0.2
	Dropout	0.5
	Momentum	0.0
	Activation Function/Architecture	ReLU, SELU
	Minibatch size	128
MF	Dimension of latent space	8, 16, 32, 64
	Precision of observation	1.0, 5.0, 10.0
	Precision of compound features	1.0, 5.0, 10.0
	Number of burn-ins	300
	Number of samples	100, 200, …, 1700
XGB	Objective	binary:logistic
	Learning rate	0.05
	Scale positive weight	1, 5, 10
	Number estimators	50, 100, 200
	Maximum depth	5, 10

Table S1 Considered machine learning algorithm hyperparameters. Hyperparameters values were systematically varied following a grid search.

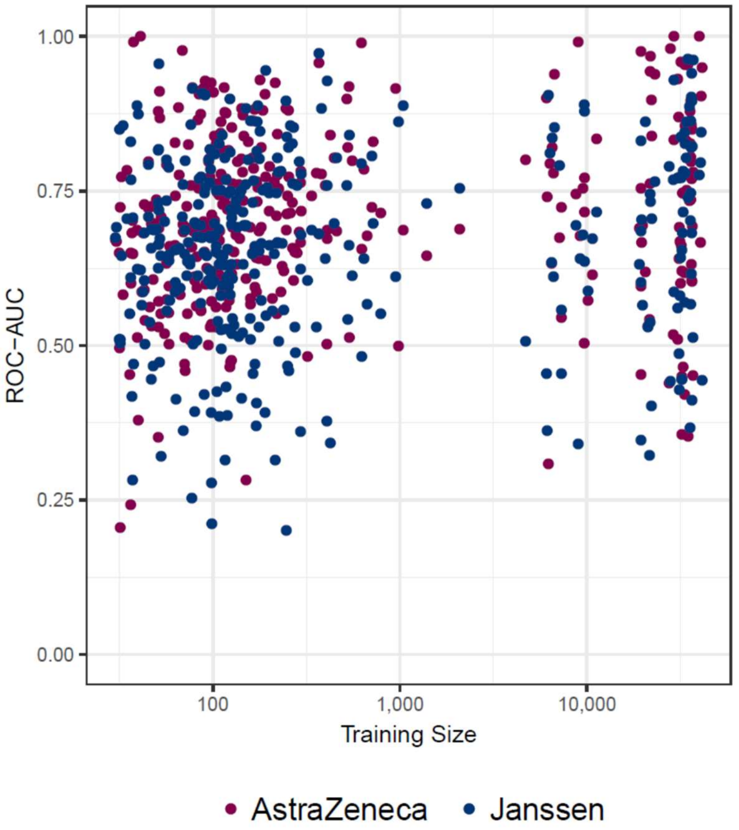

Fig. S3 DNN ROC-AUC performances on industrial datasets vs. Training set size. Each dot corresponds to a target. Only those targets, which are available in all 3 datasets (ExCAPE-ML, AstraZeneca and the Janssen dataset) are shown.
